# Potentization

**Published:** 1883-12

**Authors:** B. Fincke


### POTENTIZATION.

*To the Editor of* THE HOMŒPATHIC PHYSICIAN:

DEAR DOCTOR:—In the November number of THE HOMŒOPATHIC PHYSICIAN is an editorial entitled "Fallacious Theories," in which you attack the thesis which I had defended in your October number of last year, page 417, viz.: *Potentization makes the medicine homœopathic.*

You say: "A drug that is not homœopathic, *i. e.*, is not the most like, the simillimum to a set of symptoms in the third or thirtieth potency, becomes so if used in the cm or mm." But my thesis is: Potentization makes the medicine homœopathic, and your explanation of it means something else, is itself a fallacy, and leads to other fallacies in your further reasoning, being false in the premises. I have proved my thesis by the fact that poisons and inert substances are not homœopathic *per se*, because the former kill and the latter make no impression on the organism. Only by potentization they become homœopathically active, because only then they are enabled to meet similar symptoms curatively. The proposition, that "a drug, not being homœopathic in the third and thirtieth, becomes so in the cm and mm," is of your own invention, and, naturally, the fault you find with it recoils upon the inventor.

Furthermore, the argument in my article applied to no higher potency than the thirtieth centesimal, which Hahnemann advocated ever since the last edition of the *Organon* and the second edition of the *Chronic Diseases*, and the consideration of the higher potencies was purposely omitted; hence, the introduction of the "cm and mm" is entirely gratuitous.

"A drug," you continue, "is only homœopathic to a set of symptoms when its pathogenesis is similar to those symptoms. It is, then, homœopathic, no matter what potency it be used in." But Hydrocyanic and Silicic Acids have no pathogenesis similar to a similar set of symptoms; hence, they cannot be homœopathic and cannot be used for the homœopathic purpose of healing. They are not yet a potency, at least not a homœopathic one, and in order to fit them for homœopathic use, they must undergo the process of potentization. This, unknowingly, you admit, because you say, "No matter what potency it be used in," by which I suppose you mean, if it is no potency, it is not to be used, implying in this manner the necessity of my thesis, which, however, you pronounce fallacious.

"We," you continue, "believe the high will *always*, or *very nearly always*, cure best; but the lower potencies *do* cure. Some of our grandest cures have been made with them, and it is folly to assert that Homœopathy only begins with high and highest potencies. A wise homœopathist will not limit himself to any one potency, but will keep his mind unprejudiced, to use whichever preparation seems to be best." This is precisely the position which the great majority of the Institute has taken and has given rise to a separate body, the organ of which, THE HOMŒOPATHIC PHYSICIAN, is trusted to your editorial hands.* For one, I object to that position, and say it is anti-Hahnemannian and anti-homœopathic, because Hahnemann never advocated such a doctrine, and, besides, it contradicts the essential points which form the medical creed of the very Association you represent. To be sure, the admission in favor of "the high" is very gratifying, but the advice following it mars the good impression again, since it leads to a sentence which likewise is denounced as folly, and why? Certainly not because Hahnemann has advocated it in his *Organon* and other writings? Where did Hahnemann say "A wise homœopathist will not limit himself to any one potency, but will keep his mind unprejudiced to use whichever preparation seems to be best?" Nowhere. It is just that misunderstanding and ignoring of Hahnemann's fundamental law, that disease is only owing to the immaterial and spirit-like change of the healthy state of the organism, the distuning of it, as he terms it, which leads to the backsliding in posology, even with some of our best men. The ground-rule, similia similibus, is the rigorous criterion for posology just as well as for the selection of the remedy, because, if disease is originating from the spirit-like distunement of the organism, the remedy selected according to its homœopathicity must be prepared in such a manner that it becomes adequate to the distunement or similar to its origin—it must, in Hahnemann's words, be potentized. This is the strict postulate of Hahnemann, which he did not develop *a priori* out of his inner consciousness, but which *a posteriori* he arrived at by careful observation, correct experience, and experiment, called by Hering the inductive method. If low potencies and crude drugs cure, and even some of the grandest cures have been made by them, it by no means

* THE HOMŒOPATHIC PHYSICIAN is the organ of no association, is owned by none; it aims to represent *Homœopathy*.—EDITOR.

proves that the lowness of the potency and the crudeness were the necessary condition of their great efficacy—this was accidental—but it proves their homœopathicity, and it just as little proves that higher potencies would not have been just as effective. To see macrodosia defended in a journal, which since its existence has brought out the finest record of cures with high potencies, and by its own editor,* is not a novel sight. The same inconsistency was observable in Skinner's quarterly, the *Organon.* The position is the same as assumed by Horace M. Paine, whom I failed to convince of the Hahnemannian ideas, as early as 1864, in a voluminous correspondence, which is at your service if you wish to publish it.

But even admitting that your proposition that low potencies do cure is correct, which is not denied, you prove by the same only what the thesis sets forth; for, why do you prefer low potencies to crude drugs, if not because the medicinal power is developed, *i. e.*, because they are potentiated? Even Breyfogle and C. Wesselhœft assume as much when they limit the potentiation to the tenth and twelfth centesimal.

It is, therefore, not easy to see why you should find fault with my thesis. There is a class of remedies which stand between the poisons and inert substances which have no powerful action in their crude state. They show a low state of homœopathicity in their administration without being potentiated. But you will hardly, I think, persist in the opinion that they do not need any potentization to make them homœopathic. They, in their crude state, will be homœopathic only in a very limited range, and, compared with the extension and expansion of pathopoëtic power, which they acquire by potentization, their homœopathicity in the crude state shrinks to almost nothing; besides, too much weight has been attached to those cures with crude drugs and low potencies, as I have shown in my article.

You say: "If potentiation makes a medicine homœopathic, it must change it. Therefore, Aconite 3d, 30th, and cm are three different drugs, useful for different cases, must have different pathogeneses, and should have different names. It is this fallacious teaching which bolsters up the twin heresy of isopathy."

Experience, indeed, teaches that the various potencies of one and the same remedy exert different actions upon the organism, which, for the dogma of the anti-Hahnemannians, is very unfortunate,

---

* Never published a clinical case in my life!—EDITOR.

because it introduces an entirely new vista for medical and homœopathic treatment; but it cannot be helped, as it depends upon fact. It follows, by no means, that every potency is a separate drug by itself, which is contradictory, because they all have been developed from the same original crude drug. If you have the whole scale of potencies of a remedy, as far as it is possible to potentize it, these potencies, every one by itself having been proved upon the healthy, will, collectively, give the complete pathopoëtic picture of that remedy. Hahnemann has made the beginning, and his highest potency, proved pathopoëtically, was the thirtieth centesimal. Why did he not prove the third of Silicea or Carbo veg. or Sepia or even the crude substances? Because they could not give the clear and ample pathopoëtic picture of the thirtieth potency. If he lived to-day he would continue to prove higher potencies, not because they were higher or because he had a high-potency mania, but because they give more varied and distinct symptoms in their pathopoësis.

Your sarcasm, therefore, fails of its object, and the fallacious teaching is not on my side. Aconite is Aconite, "in the third, in the thirtieth, and in cm;" but every potency has its own characteristic, which must be considered in posology.

It remains for me to protest against the effort to couple me with those isopathists of yours, with whom I have nothing in common. At the close of the first volume of his *Chronic Diseases*, Hahnemann has distinctly laid down the rule for those substances called isopathic, or, according to Hering, nosodes. To this I adhere, and the invective comes not with very good grace, since you have published already my provings of Vaccinin, and there is a number of my provings of Variolin in your hands for future publication, from which it appears that I follow the Hahnemannian way of proving the medicines upon the healthy in order to find out their homœopathicity.

I have nowhere said that "Syphilinum is homœopathic, because it is potentized very high." Alas! that poison is already homœopathic with a vengeance, as the many infected people testify; for even in its crude state it produces a "set of symptoms" which last a lifetime and poison the future generations, if not cured. It, therefore, is a poison which needs further potentization in order to develop its curative properties. If I say, homœopathic with a vengeance; I mean only that it is an agent which poisons the system and the effects of which indicate the line of homœopathic action which it may be able to set up in the healthy organism. It seems to me, however, homœopathic is here a misnomer, if taken only for the pathopoëtic action of infection. It would be correct if this infection were considered as a set of symptoms which may lead to their cure, by the same remedy, in other infected people; a remedy which is actually not the same (ison), but only similar, according to Hahnemann, being applied upon other persons and in the potentized state; or would you, according to *your* argument, contend that syphilinum is homœopathic *per se,* without being potentized, and, hence, be obliged to advocate its application therapeutically in the crude condition, or low potency? This is the dilemma.

*Ceterum censeo macrodotiam esse debendam.*

By publishing this letter in your next number you will prevent further fallacies, and oblige, Yours, faithfully,

B. FINCKE.

